# Overview of the Anticancer Potential of the “King of Spices” *Piper nigrum* and Its Main Constituent Piperine

**DOI:** 10.3390/toxins12120747

**Published:** 2020-11-26

**Authors:** Eleonora Turrini, Piero Sestili, Carmela Fimognari

**Affiliations:** 1 Department for Life Quality Studies, Alma Mater Studiorum—Università di Bologna, corso d’Augusto 237, 47921 Rimini, Italy; eleonora.turrini@unibo.it; 2 Department of Biomolecular Sciences (DISB), Università degli Studi di Urbino Carlo Bo, Via I Maggetti 26, 61029 Urbino, Italy; piero.sestili@uniurb.it

**Keywords:** *Piper nigrum*, piperine, cancer therapy, anticancer mechanisms, in vitro studies, in vivo studies, chemosensitization, toxicological profile

## Abstract

The main limits of current anticancer therapy are relapses, chemoresistance, and toxic effects resulting from its poor selectivity towards cancer cells that severely impair a patient’s quality of life. Therefore, the discovery of new anticancer drugs remains an urgent challenge. Natural products represent an excellent opportunity due to their ability to target heterogenous populations of cancer cells and regulate several key pathways involved in cancer development, and their favorable toxicological profile. *Piper nigrum* is one of the most popular spices in the world, with growing fame as a source of bioactive molecules with pharmacological properties. The present review aims to provide a comprehensive overview of the anticancer potential of *Piper nigrum* and its major active constituents—not limited to the well-known piperine—whose undeniable anticancer properties have been reported for different cancer cell lines and animal models. Moreover, the chemosensitizing effects of *Piper nigrum* in association with traditional anticancer drugs are depicted and its toxicological profile is outlined. Despite the promising results, human studies are missing, which are crucial for supporting the efficacy and safety of *Piper nigrum* and its single components in cancer patients.

## 1. Introduction

Nowadays, cancer represents one of the biggest challenges that must be handled as a multifaceted global health issue. In fact, cancer is still the second leading cause of death worldwide and was responsible for 9.6 million deaths in 2018 [1]. Notwithstanding advances in the knowledge of cancer, supported by cutting-edge research and advanced technologies for its diagnosis and treatment, the discovery of new therapeutic agents is a hot topic in cancer research. The major disadvantages of conventional chemotherapy are the recurrence of cancer, drug resistance, and toxic effects on non-targeted tissues. Moreover, side effects can restrain the use of anticancer drugs and thus impair a patient’s quality of life [2]. Numerous medicinal plants and isolated phytochemicals have gained immense attention due to their ability to target heterogeneous populations of cancer cells and regulate key signaling pathways involved in cancer development at different stages and their wide safety profile [3].

Black pepper (*Piper nigrum* L. family Piperaceae) is one of the most used household spices in the world, with its characteristic biting quality. The use of black pepper is not limited to culinary purposes, and it is also used as a preservative, an insecticide, and medication [4]. *Piper nigrum* is a perennial climbing herb native to the Malabar Coast of India. The herb grows up to a height of 10 m by means of its aerial roots. The black pepper fruits, which are obtained from dried green unripe drupe, and seeds have been extensively used in folk medicine to treat conditions ranging from gastrointestinal diseases to epilepsy [5]. The medical properties of pepper are mainly imputable to the alkaloid piperine. Piperine exerts anti-inflammatory, neuroprotective, immunomodulatory, cardioprotective, and anticancer effects [6,7]. Moreover, piperine is well-known to influence the bioavailability of drugs and nutrients, increasing their intestinal absorption and regulating their metabolism and transport, thus representing a bioenhancer [7].

Several previous reviews have explored the potential antidepressant, antispasmodic, antidiarrheal, antiasthmatic, antimicrobial, antifungal, antioxidant, and anticancer properties of *Piper nigrum* [8,9,10,11,12]. Research on the anticancer effects of *Piper nigrum* and its constituents is a hot topic, as evidenced by the large number of recent publications on the subject, deserving an updated synopsis. The present paper provides a quite comprehensive overview of the anticancer potential of *Piper nigrum* and its main bioactive component piperine. We highlight the key mechanisms involved in the anticancer activity of piperine, with a glimpse of the activity of other bioactive molecules of *Piper nigrum* or its extracts. We also present the chemosensitizing effects of piperine in association with traditional anticancer chemotherapy and analyze and discuss its toxicological profile.

## 2. Anticancer Activity of *Piper nigrum* Extracts

*Piper nigrum* extracts could offer an interesting synergy of its single bioactive constituents, achieving anticancer activity through complementary mechanisms. Extracts from different parts of the plant, including roots, seeds, and fruits, have been explored. Different preparations of the extract from the same part of the plant give rise to different and surprising effects (Table 1), discussed hereunder. 

Ethnomedicinal surveys have revealed that seeds and fruits are the most used and studied part of the *Piper nigrum* plant [4].

Different seeds’ ethanolic extracts (50, 70, or 100% ethanol) were studied in three colorectal cell lines (Table 1). The highest cytotoxic effect was seen for the 50% seeds’ ethanolic extract (EEPN) [13]. The highest biological activity of EEPN was imputable to the highest content of total phenolic compounds extracted. Additionally, EEPN showed antioxidant and anti-inflammatory properties, which were assessed by biochemical assays [13]. No insight into the molecular mechanisms of EEPN’s cytotoxicity was provided. Recently, Tammina and colleagues [14] investigated the anticancer activity of a *Piper nigrum* water seeds’ extract formulated as SnO_2_ nanoparticles in colorectal (HCT-116) and lung (A549) cancer cell lines (Table 1). They demonstrated that higher dose and smaller size nanoparticles generated more reactive oxygen species (ROS) and hence exhibited a higher cytotoxicity compared to larger size nanoparticles [14], underlying the crucial role of formulation in improving the biological activity of *Piper nigrum* preparations.

The anticancer activity of a macerated ethanolic extract of *Piper nigrum* fruits was explored in both in vitro and in vivo breast cancer models [15] (Table 1). Treatment with the extract induced intracellular oxidative stress, which was considered the main component responsible for its cytotoxic effects in cancer cells. Since ROS can cause DNA damage, the observed oxidative DNA damage corroborated ROS involvement in the anticancer effects of the extract. These findings were confirmed in vivo, where increased lipid peroxidation and protein carbonylation and an elevated activity of the antioxidant enzymes were recorded (Table 1) [15]. The same research group investigated the anticancer potential of a high-pressure extract from unripe fruits of the black pepper cultivar Bragantina, obtained by supercritical fluid extraction (SFE) [16]. SFE represents an energy-efficient and environmentally friendly extraction technology that helps to overcome the limitation of the poor solubility of molecules such as piperine. The SFE extract showed a higher content of piperine and the highest cytotoxic activity compared to conventional ethanolic extracts [16] (Table 1). A following docking study [17] revealed the interaction of piperine with the ATP binding site of the cell-cycle regulators cyclin-dependent kinase 2 (CDK2) and cyclin A and with the antiapoptotic protein Bcl-xL. In vitro and in vivo studies on the cytotoxic activity of the SFE extract confirmed its ability to arrest the cell cycle in the G2/M phase and its pro-apoptotic effects through CDK2, cyclin A, and Bcl-xL inhibition [17]. Interestingly, treatment with macerated *Piper nigrum* ethanolic fruit extract induced cell-cycle arrest in the G1/S phase [15], whereas the SFE extract induced cell-cycle arrest in the G2/M phase [17]. This is not surprising if we consider that the SFE extract is enriched in piperine, which univocally induces cytostasis in the G2/M phase in breast cancer cells (Table 2). These data suggest that piperine is the main component responsible for the anticancer effects of the SFE extract.

Sriwiriyajan and colleagues [18] explored the cytotoxic activity of methanol and dichloromethane crude extracts of *Piper nigrum* fruits in different breast cancer cell lines (MCF-7, MDA-MB-231, and MDA-MB-468). Both extracts promoted cancer cell death. The calculated half maximal inhibitory concentration (IC_50_) (Table 1) indicated a different sensitivity to *Piper nigrum* among the three cell lines, probably dependent on their different p53 status [18]. Of note, both *Piper nigrum* crude extracts showed less marked cytotoxic effects in normal breast cells (MCF-12A), suggesting selectivity towards breast cancer cells. Interestingly, the extracts were even more cytotoxic than the two main alkaloids of *Piper nigrum* piperine and pellitorine (tested up to 20 µg/mL) in MDA-MB-468 cells. Chromatographic separation was performed to further understand which fraction, and thus which bioactive compounds, were responsible for the cytotoxic activity of the dichloromethane *Piper nigrum* extract, characterized by the best anticancer activity. Surprisingly, the isolated fractions of alkaloids without piperine, named DE and DF, showed the best IC_50_ value and significant pro-apoptotic activity [18]. However, the DE fraction lost partial selectivity versus cancer cells, as demonstrated by the extract, with a comparable induction of cell death in normal and cancer cells, while DF maintained a selective anticancer effect (IC_50_: 6.51 µg/mL in MCF-7 cells versus 20.66 µg/mL in MCF-12A).

Motivated by the anticancer activity of DE and DF piperine-free fractions, the same research group further investigated the antitumor effects of a piperine-free *Piper nigrum* extract (PFPE) [19]. The antiproliferative effects of PFPE were explored in breast, colorectal, lung, and neuroblastoma cancer cell lines (Table 1). The greatest cytotoxic effect was recorded in MCF-7 cells, where the pro-apoptotic activity of the extract was demonstrated through the p53 and cytochrome c increase, together with the induction of DNA damage via topoisomerase II downregulation (Table 1). Of note, the PFPE extract displayed a less marked antiproliferative effect in non-transformed breast cells, with a selective index (SI) value of 6.22 when compared to MCF-12A [19], thus exhibiting not only the highest anticancer effects, but also the best selectivity, in this cancer model (Table 1). The anticancer effects of PFPE were additionally investigated in Sprague-Dawley rats treated intraperitoneally with 50 mg/kg N-nitrosomethylurea (NMU)—a reliable carcinogen-alkylating agent—at 50, 80, and 110 days of rats’ age. Two different PFPE treatment regimens were administered orally to test its protective effects against NMU-induced mammary tumors in cancer initiation and post-initiation stages [19] (Table 1). In the first treatment regimen, rats were exposed to 100 or 200 mg/kg body weight (b.w.) piperine at 14 days after NMU application three times per week up to 76 days. At the end of treatment, the incidence of rats bearing tumors was 100% in the control and vehicle groups, and 20% and 10% in PFPE-orally-treated rats at 100 and 200 mg/kg b.w., respectively. In the second treatment regimen, rats were administered PFPE (100, 200, or 400 mg/kg b.w.) every two days up to 30 days after the first NMU-induced tumor was detected. Treatment with the extract significantly reduced the growth rate of tumors compared to control and vehicle groups and induced apoptosis in tumor tissues [19].

A following study [20] investigated the mechanisms underpinning the anticancer effects of PFPE in the same in vitro and in vivo breast cancer models used in [19] (Table 1). PFPE controlled the tumor size and inhibited cancer cell proliferation through the downregulation of c-myc and the upregulation of p53. Moreover, the extract had a pro-apoptotic effect mediated by PFPE pro-oxidant activity. The later stages of tumorigenesis were counteracted through (1) angiogenesis inhibition through vascular endothelial growth factor (VEGF) downregulation and (2) migration/invasion reduction via downregulation of the matrix metalloproteinase (MMP)-2 and -9 [20] (Table 1). Surprisingly, the study recorded a reduction of E-cadherin, which, in tumors, is usually associated with metastasization and cancer recurrence [22]. However, no invasion or metastasization was recorded in the study reported above [20], suggesting that the downregulation of E-cadherin induced by PFPE alone is not able to stimulate invasion and metastasis.

Of note, the role of the vehicle Tween 80 was assessed to clarify its contribution in the anticancer mechanisms evoked by PFPE [20]. Tween 80 helped to dissolve PFPE and to contrast the pungent taste, favoring oral administration in rats. No significant toxic or anticancer effects were recorded after treatment with the vehicle alone. However, Tween 80 may enhance drug uptake, increasing the cell membrane permeability thanks to its chemico-physical characteristics of nonionic surface-active detergent [20]. Therefore, the contribution of the vehicle may not be disregarded in the overall biological effects of the extract.

Only one study has explored the anticancer potential of a root’s extract. Ee and colleagues [21] investigated the cytotoxic activity of three different crude extracts of *Piper nigrum* roots: (i) chloroform; (ii) petroleum ether; and (iii) ethyl acetate followed by ethanol extraction solution (Table 1). The anticancer activity was higher for the chloroform extract (IC_50_: 9.8 µg/mL) than for the petroleum ether extract (IC_50_: 11.2 µg/mL), whereas the ethyl acetate extract showed no cytotoxic activity in human promyelocytic leukemia cells [21]. The study analyzed each extract for its alkaloid content. The biological activity of the petroleum ether extract was ascribed to the piperine content and that of the chloroform extract was ascribed to the presence of a mixture of other alkaloids, such as cepharadione, piperlactam, and paprazine [21], for which the anticancer activity has not been assessed in any other studies to date.

Taken together, these results suggest that *Piper nigrum* extracts, where several bioactive molecules coexist, may represent a promising strategy for contrasting cancer in the first and later stages of its development.

## 3. In Vitro and In Vivo Anticancer Activity of Piperine

*Piper Nigrum* represents an attractive source of bioactive compounds, such as the noteworthy piperine. Piperine, which is chemically known as 1-piperoyl piperidine, is an alkaloid found in both *Piper nigrum* and *Piper longum*, and is responsible for the pungency of pepper. Pungency results from the activation of the ion channels’ transient receptor potential cation channels for vanilloid (TRPVs). Usually, piperine is isolated and purified from pepper fruits and roots; however, its broad biological potential inspired new strategies for its biosynthesis, such as the use of endophytes. For instance, endophytic fungi isolated from *Piper nigrum* or *Piper longum* represent an alternative source of metabolites present in the host plants, such as piperine, and the optimization of their culture conditions may allow large-scale biosynthesis of this alkaloid [23,24]. Several studies have investigated the anticancer effects of piperine in in vitro and in vivo models and explored the mechanisms underpinning its anticancer activity (Table 2), both alone and in association with anticancer drugs.

### 3.1. In Vitro Studies

#### 3.1.1. Piperine Induces Apoptosis and Inhibits Cell Proliferation in Cancer Cells

##### Induction of Cell Death

Several papers have demonstrated that piperine activates caspase-3 (Table 2)—a downstream effector caspase [58]—and its reporter poly(ADP-ribose) polymerase (PARP) in breast [26], prostate [33], colon [38], melanoma [47], and ovarian [50] cancer cell lines (Table 2). Many papers have reported the activation of apoptotic final effectors such as caspase-3 by piperine, but less of them have carefully analyzed the upstream targets leading to this event.

Survivin is highly expressed in human cancers, where it is involved in the regulation of cytokinesis and cell-cycle progression, and participates in a variety of signaling pathways, including apoptosis [59]. Survivin is an anti-apoptotic protein whose expression is induced by Akt signaling. Interestingly, piperine inhibited the expression of survivin and promoted the activation of pro-caspase-3 and -7 in colon cancer cells [38]. The treatment of colon cancer cells with the inhibitor of survivin—YM-155—reduced the pro-apoptotic activity of piperine [38], suggesting that its anticancer activity depends on survivin’s expression. Accordingly, the inhibition of both survivin and phosphorylation of its transcription factor p65 was observed in human and murine breast cancer cells after piperine treatment [27], highlighting that survivin downregulation prompts piperine pro-apoptotic effects in different cancer types.

Apoptosis can be executed by promoting two major pathways: the mitochondrial-mediated intrinsic pathway and/or the death-receptor-mediated extrinsic pathway. The mitochondrial pathway often represents an entry point for the activation of caspases via the release of apoptogenic factors such as cytochrome c, the second mitochondria-derived activator of caspase/direct inhibitor of apoptosis protein (IAP)-binding protein with low PI (Smac/DIABLO), and the apoptosis-inducing factor (AIF) into the cytosol [58]. Greenshields and colleagues [28] demonstrated that piperine impacts the intrinsic apoptotic pathway via SMAC/DIABLO regulation in breast cancer cell lines with basal human epidermal growth factor receptor 2 (HER2) expression. The regulation of this pro-apoptotic pathway by piperine was confirmed in melanoma cells [47,48], where piperine downregulated the anti-apoptotic protein human X-linked IAP (XIAP), representing the most characterized among the IAP proteins [47]. Within the overarching frame of apoptosis [58], these results suggest that piperine may orchestrate Smac/DIABLO binding of XIAP, allowing the displacement of caspase-9 from XIAP and, in turn, the formation of the cytochrome c/Apaf-1/caspase-9-containing apoptosome complex.

The upregulation of Bax and the downregulation of Bcl-2 further confirmed the pro-apoptotic activity of piperine mediated by the intrinsic pathway in melanoma, hepatocarcinoma, breast, lung, prostate, leukemia, and ovarian cancer cells [30,36,45,48,49,51,57] (Table 2). Bax and Bcl-2 do not represent the only proteins belonging to the Bcl-2 family regulated by piperine. Indeed, piperine downregulated the uncleaved BH3 interacting domain death agonist (Bid) in melanoma cells [47]. Bid may represent a point of interconnection between intrinsic and extrinsic pathways, because its cleavage by caspase-8 allows the release of cytochrome c and the mitochondrial amplification of caspases [58]. However, no studies have demonstrated the activation of caspase-8 by piperine. Moreover, the pre-treatment of ovarian cancer cells with specific caspase-3 and -9 inhibitors blocked the apoptosis induced by piperine, while the caspase-8 inhibitor did not affect the pro-apoptotic effects of piperine [50], suggesting no activation of the extrinsic pathway.

Piperine increased the expression of p53 in melanoma and lung cancer cells [45,47]. However, Greenshields and co-authors showed that the anticancer effects of piperine did not require a functional p53. They also demonstrated the cytotoxic effects of piperine in p53-deficient breast cancer cells [28]. Similar results were reported in colon cancer, where piperine significantly inhibited cell growth in p53 −/− HCT116 cells [38]. Since p53 is often mutated or not functional in many kinds of tumors [60], the pro-apoptotic activity of piperine independent from p53 may have clinical relevance.

The expression of fatty acid synthase is closely related to aggressiveness and cell-cycle progression in human cancers, such as breast cancer [61]. Interestingly, piperine suppressed the expression of fatty acid synthase through the inhibition of extracellular signal-regulated kinase 1/2 (ERK1/2) and the reduction of mature sterol regulatory element-binding protein-1 (SREBP-1), leading to apoptosis in HER2-overespressing breast cancer cells [26].

G-quadruplex DNA structures are non-canonical DNA structures generated by square planar arrangements of G-quartets and are key regulators of cellular processes, such as replication, transcription, and translation [62]. The majority of G-quadruplex structures are generated in telomers and in the regulatory regions of oncogenes, such as c-myc, contributing to maintaining shorter telomers and causing genome instability, finally leading to cancer development [63]. A recent publication showed that piperine bound with a high affinity to a G-quadruplex structure formed in the c-myc promoter region (Pu24T) in lung cancer cells [46] (Table 2). The stabilization of this G-quadruplex structure led to a reduction of the c-myc expression in cancer cells [46], representing a candidate mechanism behind the pro-apoptotic activity of piperine.

Several natural compounds exhibit anticancer effects via autophagy-regulating activities. Autophagy represents a double-edge sword that, in cancer cells, can either suppress or promote tumorigenesis [64]. In addition to apoptosis, piperine-induced autophagy was demonstrated in prostate and leukemia cancer cells [32,57]. For instance, piperine increased the autophagosomal markers of the microtubule-associated protein 1A/1B-light chain 3-phosphatidylethanolamine conjugate (LC3B-II) and the formation of LC3B puncta in prostate cancer cells [32].

Prostate cancer, especially in the early-stages, depends on androgens for growth and survival. Accordingly, androgen ablation therapy results in cancer regression [33]. Irrespective of the apoptotic or autophagic cell death induced by piperine in prostate cancer cells [32,33,34], several studies agree on identifying androgen-dependent cells (LNCaP cells) that are more susceptible to the anticancer effects of piperine than androgen-independent prostate cancer cells (PC3 and DU145) (Table 2). The higher sensitivity of androgen-dependent cells may be due to piperine’s capacity to downregulate the expression of androgen receptors that in turn contributes to blocking prostate cancer progression. This hypothesis is consistent with the reduction of prostate specific antigen (PSA) levels—a marker for prostate cancer progression—observed after piperine treatment in LCNaP cells [33]. Taken together, these results highlight that piperine may also represent a promising anticancer strategy in androgen-dependent prostate cancer.

##### Pro-Oxidant Activity and Induction of Endoplasmic Reticulum (ER) Stress

Piperine exhibits antioxidant activity at low doses [65], while at higher doses, it stimulates the production of ROS in many cancer cells (Table 2). Due to their role in favoring the survival or death of cancer cells, ROS are a double-edge sword [66]. Oxidative stress caused by an excess of ROS, including superoxide, hydrogen peroxide, and hydroxyl radicals, is a well-known inducer of apoptosis [67]. Therefore, the pro-oxidant activity of piperine may contribute to its ability to induce apoptosis in many cancer models [38,41,47,49,55,56]. In liver cancer cells, the intracellular accumulation of peroxides by piperine resulted in cytosolic pH acidification that triggered Bax oligomerization and Bcl-2 inactivation and apoptosis [49]. Moreover, oxidative stress is linked to damage to nucleic acids [68]. In melanoma cells, piperine was shown to generate ROS in a concentration-dependent manner and DNA double-strand breaks. Interestingly, pre-treatment with the antioxidants tiron and N-acetyl-L-cysteine (NAC) prevented the piperine-induced DNA damage and its cytotoxic and cytostatic effects [47]. Accordingly, piperine induced apoptosis by an increase of hydroxyl radicals in human rectal adenocarcinoma cells and treatment with NAC suppressed its pro-apoptotic effects [41]. These results depict a direct link between ROS generation and DNA damage induced by piperine, finally leading to cell-cycle arrest and apoptosis. However, Greenshields and colleagues [28] showed that, in a breast cancer cell model, apoptosis by piperine might be independent of ROS formation. In particular, they demonstrated the inability of pre-treatment with glutathione (GSH)—one of the main intracellular defenses against oxidative stress—to reduce the fraction of apoptotic cells after piperine exposure. Moreover, they observed synergistic cytotoxic activity between piperine and ionizing radiations, speculating that synergy resulted from the combination of the ROS-dependent cytotoxic mechanism of radiation and ROS-independent cytotoxic mechanism of piperine. Furthermore, Yaffe and colleagues [38] showed that the anticancer effects of piperine were partially dependent on ROS generation in colon cancer cells. They demonstrated that pre-treatment with NAC partially protected colon cancer cells from early apoptosis, but did not reduce late apoptotic cell death. On the whole, the studies reported above suggest that the anticancer mechanisms evoked by piperine may not be completely ascribable to oxidative stress and may be cell-type specific, prioritizing one pathway over the others.

Yaffe and colleagues demonstrated that piperine induces endoplasmic reticulum (ER) stress in colon cancer cells [38]. In particular, piperine treatment triggered the unfolded protein response and upregulated the ER stress-associated proteins such as inositol-requiring 1α (Ire1α), C/EBP homologous protein (CHOP), binding immunoglobulin protein (Bip), and conceivably protein kinase R-like ER kinase (PERK), promoting caspases’ activation and apoptosis [38]. Interestingly, ER stress and oxidative stress form the engine that could promote a special kind of regulated cell death referred to as immunogenic cell death (ICD). ICD activates an adaptative immune response in the host, representing the link between tumor demise and recruitment of the immune system to promote anticancer immunity [69]. Although many further studies are required to identify piperine as a valuable ICD inducer, its ability to promote ER stress and ROS generation is the requisite starting point for exploring this possibility.

##### Induction of Cell-Cycle Arrest

The antiproliferative activity of piperine has been recorded in many types of cancer cells (Table 2). According to the cell type and tumor behavior, piperine can arrest the cell cycle in G0/G1, S, or G2/M phases. Piperine arrested the cell cycle in the S phase only in leukemia cells [57], whereas it halted the cell cycle in the G1 phase in colon [38], melanoma [47], and prostate cancer cells [32,34,35]. The block in G1 was associated with the downregulation of cyclin D and upregulation of p21 (Table 2). P21 is an inhibitor of cyclin-dependent kinases (CDKs). Moreover, it blocks the phosphorylation of the retinoblastoma protein (pRb) and attenuates the expression of E2F Transcription Factor 1 (E2F1), thus inhibiting the progression of the cell cycle into and through the S phase [70]. P21 was also found to inhibit the activity of cyclin A and B, which are necessary for the progression through S and G2/M phases, respectively. The overexpression of p21 and the cell-cycle block in the G2/M phase were observed after piperine treatment in breast [25,27,28], ovarian [51], lung [45], osteosarcoma [52], oral squamous carcinoma [55], and cervical adenocarcinoma cancer cells [56] (Table 2). Interestingly, in a mammalian breast cancer model, piperine had a broad inhibitory effect on both G1/S and G2/M transition, confirmed by the upregulation of cell division cycle 25C (CDC25C) [28], which is a phosphatase involved in both of the aforementioned transitions, and the downregulation of cyclin B, but not cyclin D [25].

Together with the overexpression of p21, piperine induced p27 upregulation, which can influence the cell cycle in several ways. For instance, p27 blocks the activity of CDK4-cyclin D and CDK6-cyclin D, causing cell-cycle arrest in the G1 phase. Fofaria and colleagues [47] demonstrated that G1 arrest by piperine was linked to DNA damage induced by the pro-oxidant activity of piperine and activation of the checkpoint kinase Chk1. DNA damage may activate proteins such as ataxia-telangiectasia-mutated/ataxia telengectasia and Ras3-related proteins (ATM/ATR) that in turn phosphorylate checkpoint kinases Chk1 and Chk2 [71]. The block of Chk1 activation by a specific inhibitor or the use of siRNA protected melanoma cells from piperine-induced cell-cycle arrest [47]. This suggests the crucial role of Chk1 phosphorylation in piperine-mediated G1 inhibition. In osteosarcoma cells, piperine treatment blocked cells in the G2/M phase, which is an event associated with Chk2 phosphorylation [52]. These latter studies clearly depict the temporal sequence of events evoked by piperine: activation of the ATR/Chk/p53/p21 axis finally leading to cell-cycle arrest and apoptosis.

Taken together, these results disclose that cell-cycle regulators targeted by piperine strongly depend on the cancer cell type and determine the phase where the cell cycle is blocked.

3D cell cultures represent a promising bridge between traditional monolayer cell culture and animal experiments, better mimicking in vivo tumor characteristics compared to bidimensional cell cultures. In the last decades, 3D models have seen a rapid advancement and several techniques have been proposed for obtaining 3D in vitro cancer models, i.e., multicellular tumor spheroids [72]. The main advantage of 3D cancer models is their ability to reproduce the tumor microenvironment and the presence of cancer stem cells (CSCs) [72], whose uncontrolled self-renewal is often responsible for cancer relapses and the onset of chemoresistance [73]. Piperine inhibited the growth of breast cancer spheroids, named mammospheres, and colon cancer spheroids [28,38]. The antiproliferative effect of piperine in mammospheres, exhibiting typical cancer stem cell markers [74], was significant, although less marked than in breast cancer monolayer cell cultures [28]. Several aberrant pathways are involved in the maintenance of CSC self-renewal, i.e., Hedgehog, Notch, and Wnt [75]. Piperine (5–10 µM) inhibited breast CSC self-renewal though downregulation of the Wnt signaling pathway [76]. These results highlight piperine as an important regulator of CSCs’ aberrant proliferation, thus further contrasting a key feature of different tumors.

Voltage gated K^+^ channels play an important role in regulating cell-cycle progression through the early G1 phase [77], thus representing an interesting target for the inhibition of cancer cell proliferation. Piperine significantly inhibited the voltage gated K^+^ current (Ik) in a dose-dependent fashion in prostate cancer cell lines [34,35] (Table 2), resulting in cell-cycle block in the G0/G1 phase and apoptotic cell death. However, the role of K^+^ channels in cancer progression is not entirely understood [78]. For this reason, together with the complex role of K^+^ channels in a variety of tissues, K^+^ channels’ inhibitors may represent a seducing anticancer strategy, but their use in vivo still deserves further investigation before achieving clinical relevance.

#### 3.1.2. Piperine Inhibits Cancer Metastasization and Neoangiogenesis

##### Inhibition of Invasion/Migration and Epithelial Mesenchymal Transition

The ability to invade surrounding tissues and metastasize are key hallmarks of malignant cells. Metastasis is a complex process that requires specific steps, including decreased cell adhesion, increased cell motility and invasion, resistance to apoptosis, and proteolysis via matrix metalloproteinases (MMPs) [79]. MMPs are the key proteases involved in the degradation of extracellular matrix molecules in the basement membrane, which represents the main barrier between cancer cells and the bloodstream. Piperine reduced MMP expression and activity in several cancer cell lines (Table 2). For instance, in breast cancer cells, piperine inhibited MMP-2 and MMP-9. The inhibitory effect of piperine on breast cancer cells’ migration relied not only on the direct inhibition of MMPs, but also on the downregulation of HER2 expression [26], which is a key regulator of the metastatic potential of breast cancer cells that promotes the expression of MMP-2/9 [80]. The inhibition of MMP-2 and -9 by piperine also depended on the inhibition of Akt signaling pathways, as shown in breast, osteosarcoma, prostate, and fibrosarcoma cancer cells [26,28,36,52,54]. The same findings were confirmed in mouse mammary carcinoma cells [25], where piperine downregulated the expression of MMP-13, usually expressed in invasive breast cancer cells [81]. The expression of MMPs is regulated by tissue inhibitors of metalloproteinases (TIMPs) [82]. In osteosarcoma cells, piperine increased the expression of TIMP1 and -2 that in turn inhibited MMP-2/9, further contrasting metastasization [52].

The signal transducer and activator of transcription 3 (STAT-3) is an antiapoptotic protein playing an important role in cancer cell proliferation, invasion, and migration [83]. In prostate, gastric, and colon cancer cell models, piperine inhibited migration via STAT-3 downregulation [33,40,84] (Table 2), resulting in inhibition of metastasization.

Epithelial mesenchymal transition (EMT) allows epithelial cells to differentiate into mesenchymal ones and is directly related to the improvement of migratory properties of cancer cells [85]. Da Fonseca and colleagues [31] investigated whether piperine may control transforming growth factor beta 1 (TGF-β1), which is a potent EMT inducer, in A549 lung cancer cells. TGF-β1 prompts A549 cells to lose their epithelial characteristics and to acquire a spindle-like mesenchymal morphology [86]. Pre-treatment with piperine at non-toxic concentrations inhibited the expression of mesenchymal markers induced by TGF-β1 treatment, leading to (i) the inhibition of phenotypic mesenchymal alteration, fibronectin, and N-cadherin expression; (ii) the promotion of epithelial marker E-cadherin expression; (iii) decreased cell motility and MMPs’ expression. Furthermore, piperine downregulated ERK and small mother against decapentaplegic (SMAD) signaling pathways [31,49], which represent upstream events of TGF-β1-induced EMT. The inhibition of cancer cells’ migration by piperine via EMT suppression was recently demonstrated in colon cancer cell models [40], where piperine reduced the phosphorylation of STAT-3 and the expression of the EMT regulator Snail, whose inhibition is accompanied by upregulation of the epithelial marker E-cadherin and downregulation of the mesenchymal marker vimentin [40] (Table 2).

Wnt/β-catenin signaling is a highly conserved pathway involved in the regulation of key cellular processes, such as proliferation, self-renewal, differentiation, apoptosis, and cell migration [87]. The activation of Wnt signaling is mediated by its core component β-catenin [87]. Aberrant Wnt signaling occurs in numerous cancers. For this reason, its regulation is an attractive target for cancer therapy. Very recent studies reported that inhibition of the Wnt/β-catenin signaling pathway mediates the anticancer effects of piperine in colorectal [39] and osteosarcoma cancer cells [53] (Table 2). In particular, the studies disclosed the central role of β-catenin suppression for the inhibition of Wnt signaling in cancer cells [39]. The downregulation of β-catenin downstream targets (cyclooxygenase-2 (COX-2), cyclin D1, and c-myc) triggered apoptosis and inhibited cell proliferation and metastasis [53].

Taken together, these results show that piperine inhibits cancer metastasization independently of the cancer cell type and mainly via the downregulation of MMPs’ expression and EMT inhibition. New insights suggest Wnt/β-catenin inhibition as a candidate mechanism of piperine’s anticancer effects, but further studies are needed to confirm the entity of its contribution.

##### Inhibition of Angiogenesis

Angiogenesis represents a crucial step in tumor progression that allows tumors growth [88]. Accordingly, antiangiogenic drugs have entered the clinical armamentarium against cancer. Doucette and colleagues investigated the effect of piperine on the different steps of the angiogenetic process [89]. Firstly, the authors showed that piperine inhibited proliferation and the G1/S transition of human umbilical vein endothelial cells (HUVECs), without causing cytotoxic effects. Afterwards, they demonstrated that piperine inhibited collagen-induced blood vessel outgrowth in ex vivo and in vivo models (rat aorta angiogenesis model and chick embryo CAM assay, respectively) [89]. The mechanism underpinning the antiangiogenetic effects of piperine lay in the downregulation of the pro-angiogenetic Akt signaling cascade by piperine [89]. Moreover, piperine decreased the expression of VEGF in osteosarcoma [53] and mouse breast cancer cells [29] (Table 2), further supporting its ability to interfere with the process of neoangiogenesis.

### 3.2. In Vivo Studies

Despite the abundance of in vitro studies exploring the anticancer potential of piperine and depicting the involved mechanisms, less studies have investigated its anticancer activity in animal models (Table 2). The first in vivo evidence emerged about 20 years ago and explored the ability of piperine to inhibit lung metastasis induced by melanoma cells in C57BL/6 mice [42] (Table 2). An injection of B16F-10 melanoma cells into the lateral tail vein induced lung metastasis in all treated mice. During lung metastasis, lung fibrosis occurs [42]. The extent of lung fibrosis during metastasis correlates with the content of the lung collagen hydroxyproline, massively deposited in the alveolus of the lungs. An accumulation of hydroxyproline resulted from a high content of uronic acid, produced by tumor cells as a result of aldoses’ oxidation of sugar derivatives [42]. Uronic acid leads to the formation of glucuronic acid lactone that contributes to the conversion of prohydroxyproline in hydroxyproline. Hexosamine is another sugar derivative present in tumor cells, playing an important role in the synthesis of sialic acid, which is a component of tumor cells’ surface and whose levels directly correlate with metastasis and a poor prognosis [90]. Rapidly growing tumors need intracellular GSH in order to obtain energy and sustain tumor growth and dissemination. Gamma glutamyltranspeptidase (GGT) catalyzes intracellular GSH synthesis by the gamma glutamyl cycle, representing a good marker for cell proliferation and metastasization [91]. Piperine administration (200 μmol/kg b.w. injected intraperitoneally for 10 days) significantly reduced tumor nodule formation and increased the survival rate from 31 days up to 90 days in piperine-treated mice. Moreover, piperine reduced the biochemical markers of lung metastasized tissue, including collagen hydroxyproline, lung uronic acid, and hexosamine content. In addition, piperine decreased the serum level of sialic acid and GGT, highly expressed in tumor-bearing mice [42] (Table 2). Despite the positive outcome observed in this study, significant gaps emerged due to the lack of the evaluation of the safety profile of piperine, even if an increase in mice survival was recorded up to the end of the experiments (90 days) [42].

Later studies tested whether piperine may contrast lung cancer development in male albino mice treated with the carcinogenic polycyclic aromatic hydrocarbon benzo(a)pyrene (B(a)P) twice per week for 4 consecutive weeks [43]. B(a)P is able to generate high amounts of free radicals, which react with lipids causing lipid peroxidation and with residues of proteins generating carbonyls [43,92]. Two different treatment regimens were proposed for piperine administration: (i) to study piperine effects in the initiation phase of carcinogenesis, animals received 50 mg/kg b.w. os piperine on alternate days for 16 weeks immediately after the first dose of B(a)P, and (ii) to assess the piperine effects in the post-initiating phases of carcinogenesis, animals received piperine starting from the sixth week of B(a)P treatment till the end of the experiment [43] (Table 2). The experimental design included a group receiving piperine alone for 16 weeks to assess, if any, the toxicological effects of piperine. Piperine treatment significantly decreased lipid peroxidation and the amount of protein carbonyls. Moreover, piperine reduced polyamine synthesis, which is usually elevated in body fluids of tumors bearing animals [93]. These effects may be justified by the enhancement of antioxidant enzymes and seleno proteins by piperine that may reduce carcinogen–DNA interactions and protect cells from oxidative damage. In a following study, the same authors [44] confirmed that piperine, in both treatment regimens described above, reduced hexose, hexosamine, and sialic acid in serum, lung, and liver tissues (Table 2). The protective effects of piperine were more pronounced in the initiation phase of carcinogenesis than in the post-initiation phases. This may be imputable to the downregulation of phase I enzymes and upregulation of glutathione metabolizing enzymes evoked by piperine [65], resulting in improved carcinogen detoxification.

More recently, the anticancer effects of piperine were investigated in Wistar albino rats afflicted by hepatocarcinoma induced by diethylnitrosamine (DEN), which is an extremely potent liver genotoxic carcinogen in rats [49] (Table 2). Piperine (os, 5 mg/kg b.w.) significantly decreased enzyme biomarkers of liver toxicity, such as aspartate transaminase (AST), alkaline phosphatase (ALP), and alanine transaminase (ALT) and improved the liver architecture compared to DEN-treated rats. Furthermore, piperine decreased the number of cells expressing Ki67—a proliferation marker—and increased the fraction of apoptotic cells compared to DEN-treated mice, thus supporting the inhibition of tumor growth by piperine. Interestingly, in the same experimental model of DEN-induced hepatocarcinoma rats, co-treatment with piperine and the antioxidant EUK 134, which mimics catalase activity, markedly reduced the anticancer potential of the phytochemical [49]. Therefore, this study confirmed in vivo that the main mechanism underpinning the anticancer effects of piperine may lie in its pro-oxidant activity.

Additionally, piperine (100 mg/kg/die injected intraperitoneally for 1 month) significantly reduced tumor growth in nude mice xenotransplanted with androgen-dependent (LCNaP) or androgen-independent (DU145) prostate cancer cells [33] (Table 2). The reduction of tumor growth was 72% for LNCaP-xenotransplanted mice and 41% for the DU145-xenotransplanted group. These results are in agreement with those obtained in in vitro models, where piperine was more efficient in inhibiting the proliferation of androgen-dependent prostate cancer cells [32,33] (Table 2). Moreover, for androgen-dependent tumors, the anticancer effects of piperine were confirmed in LCNaP-xenotransplanted nude mice after oral gavage administration (10 mg/kg b.w. for 1 month) (Table 2).

Piperine also exhibited anticancer effects on in vivo breast cancer models [29] (Table 2). In particular, the intraperitoneal administration of piperine (25 mg/kg/day for 14 days) in Balb mice implanted with mouse mammary EMT6/P cancer cells significantly decreased the tumor size (decrease of 15% compared to the negative control that exhibited an increase of the tumor size of 79%) [29]. Moreover, in a mouse 4T1 mammary carcinoma model, an intratumoral injection of piperine (2.5 or 5 mg/kg every 3 days three times) dose-dependently suppressed primary 4T1 tumor growth. The antitumor effect was due to its pro-apoptotic and antiproliferative effects recorded in mice tumor tissues [25] (Table 2). The inhibition of tumor growth was shown in the same breast cancer model after the oral administration of piperine (50 mg/kg/day) [27]. In this case, piperine was administered at a concentration 10 times higher compared to the intratumoral injection. This is likely due to the different pharmacokinetic pathway that piperine passes through before reaching tumor tissue after ingestion.

Since 4T1 cells can metastasize to various organs, such as the lung, this model was used to assess the in vivo the potential inhibitory effect of piperine on metastasization [25]. Piperine-treated mice, at the highest tested dosage (5 mg/kg), significantly inhibited the formation of lung metastasis, thus confirming the in vitro antimetastatic potential of piperine.

Taken together, in vivo evidence supports the anticancer activity of piperine and confirms in vitro results on its (i) pro-oxidant and pro-apoptotic activity, (ii) androgen-dependent anticancer activity, and (iii) antimetastatic potential.

### 3.3. Selectivity of Piperine towards Cancer Cells

One of the main limits afflicting anticancer drugs is their inability to selectively target cancer cells, eliciting frequent and severe toxic effects. Several pre-clinical studies compared the antiproliferative and cytotoxic effects of piperine in cancer cells versus non-transformed ones, showing at least partial selectivity of the phytochemical (Table 3).

For instance, no antiproliferative effects were observed in human lung fibroblasts treated for 48 h up to 400 µM [45] compared to lung cancer cells (IC_50_ in A549: 122 µM). On normal prostate epithelial cells, piperine did not show any cytotoxic effect up to 80 µM compared to two of the three tested prostate cancer cell lines [32], exhibiting partial selectivity. Furthermore, the IC_50_ on murine fibroblasts after 48 h of piperine treatment was more than two times higher than that observed in mouse mammary carcinoma cells (232 versus 105 µM, respectively) [25]. Piperine showed considerably weaker antiproliferative effects on normal human fibroblasts and human mammary epithelial cells compared to colon cancer cells in the range of concentration of 75–150 µM [38]. Accordingly, piperine treatment up to 200 µM did not affect the cell viability of intestinal cells compared to three different colon cancer cell lines [39]. The limited antiproliferative effects of piperine on normal cells were also confirmed in normal human mammary epithelial cells compared to four different breast cancer cell lines at a concentration of up to 150 µM [28]. Moreover, the treatment of human hFOB osteoblasts with piperine (up to 200 µM, 72 h) had a weaker growth inhibitory effect compared to that recorded in human osteosarcoma cells [52]. No significant toxicity was observed below 100 µM piperine on normal rat liver hepatocytes after 48 h from treatment [49]. Recently, Si and colleagues [50] demonstrated that piperine did not significantly affect the cell viability up to 72 h exposure at 20 µM in normal ovarian cells compared to ovarian cancer cells, where the viability significantly decreased already after 48 h piperine exposure at 8 µM.

Cancer cells are characterized by a constitutive high production of ROS compared to normal cells, which promotes their proliferation. An amount above an intolerable threshold may induce cell-cycle block and/or apoptosis [94]. Therefore, increasing ROS levels may represent a therapeutic strategy to increase the killing of cancer cells and the biochemical basis of piperine’s selectivity for cancer cells may lie in its ability to increase basal ROS generation, which differently affects cancer and normal cells.

### 3.4. Basic Aspects of Piperine Pharmacokinetics

The piperine content in black pepper is estimated to be 5–9% and a daily consumption of 0.33 g black pepper by a 60 kg person results in a piperine intake of 16.5–29.7 mg [95]. Piperine is a weak base that, after oral ingestion, undergoes hydrolysis and is characterized by a low solubility in water. Information about piperine absorption and metabolism is mainly obtained from animal studies and indicates easy intestinal absorption [10,11]. In particular, male albino rats exposed to 170 or 85 mg/kg piperine intraperitoneally displayed 97% piperine intestinal absorption and 3% excreted in feces. Additionally, the extensive hepatic metabolic conversion of piperine resulted in the urine excretion of piperonylic acid, piperonyl alcohol, piperonal, and vanillic acid and the fecal excretion of piperic acid. Following absorption, piperine distributed throughout various tissues, including the liver, kidney, spleen, stomach, and small intestine [96]. Later studies supported the rapid absorption of piperine in the gastrointestinal tract, detecting piperine in plasma after just 15 min from the oral administration of 20 mg/kg b.w. in rats [97]. Piperine could be detected in plasma up to 8 h, with the maximum plasma concentration being reached 30 min post dosing [97]. Recently, piperine’s pharmacokinetic profile was characterized after oral (20 mg/kg) and intravenous (10 mg/kg) single dose administration in Wistar rats [98]. The maximum serum concentration (Cmax) after oral administration was 0.983 µg/mL, which occurred approximately 2 h post-dose. The area under the curve (AUC) was 7.53 µg·h/mL after oral administration and 15.6 µg·h/mL after intravenous administration. Piperine achieved an extensive distribution in the body (apparent steady state volume of distribution: os 4.692 L/kg, iv 7.046 L/kg). The absolute oral bioavailability of piperine was found to be 24% [98]. Although suggesting a non-linear pharmacokinetics of piperine in combination with the anticancer drug docetaxel, a recent study in Sprague-Dawley rats fully agreed on the percentage of oral piperine bioavailability (25.36% after 3.5 mg/kg piperine) [99].

The pharmacokinetics of piperine was also assessed in healthy volunteers [100,101]. Jumpa-ngern and colleagues explored the pharmacokinetics of Benjakul formulation, representing a preparation from Thai traditional medicine whose major active component is piperine [101]. Although piperine used in the preparation was isolated from *Piper* species different from *Piper nigrum*, we report this study as one of few reporting piperine pharmacokinetics in humans. Twenty healthy subjects (10 males and 10 females, aged 20–38 years, weighing 42 to 84 kg, with a body mass index between 17.9 and 25.9 kg/m^2^) received 100 (6 mg piperine) or 200 (12 mg piperine) mg Benjakul tablets in a single oral dose [101]. No other concurrent drugs or alcohol were allowed during the study period. Meals with no composition of the plant materials present in the formulation were provided to all subjects during the investigation period. Whole blood (5 mL) was collected from each patient and placed in sterile heparin tubes 0.5, 1, 2, 4, 6, 9, 12, 18, 24, and 48 h after drug administration. The study showed that piperine’s pharmacokinetics was not dose-dependent, confirming the data obtained in a study on rats [99]. After 200 mg Benjakul administration, Cmax was 1078 ng/mL of piperine, which was significantly higher than that obtained after a 100 mg dose (467 ng/mL). The median time required to obtain these Cmax values was about 1 h. Moreover, AUC 0–48 h was greater after 200 mg administration compared to 100 mg (10.216 versus 4.288 ng·h/mL). Both Cmax and AUC values were increased by about two-fold when the dose was 200 mg compared to 100 mg, but other pharmacokinetic parameters could not be determined with accuracy (i.e., total clearance, half-life, and volume of distribution), due to the limitation of blood sampling [101].

Taken together, these data show that a limited bioavailability, but good tissue distribution, characterize piperine’s pharmacokinetics. In order to improve the bioavailability of this phytochemical, new formulations have been designed, i.e., nanoparticles, liposomes, and microtubules, for piperine alone or in association with other drugs, as described in the paragraph below. However, further pharmacokinetic studies are needed and would be useful for better predicting the efficacy of piperine as an anticancer agent.

### 3.5. Chemosensitizing Activity of Piperine

Chemoresistance has been recognized as one of the largest hurdles facing anticancer regimens. After being exposed to one anticancer drug, cancer cells can develop resistance to various other antineoplastic agents through the phenomenon called multidrug resistance (MDR) [102]. This resistance can be attributed to complex mechanisms, such as enhanced drug efflux or drug metabolism. For instance, in cancer cells, the overexpression of the efflux pump P-glycoprotein (P-gp), which is an ATP-dependent transmembrane protein belonging to the ABC transporter family, reduces the accumulation of anticancer drugs inside the tumor cell [102]. Apart from the mechanisms involved in MDR development, alterations of apoptosis’ control at different levels is crucial for the induction of chemoresistance [103].

The mechanism of MDR could be bypassed using agents defined as chemosensitizers. These substances can improve an anticancer drug’s bioavailability and efficacy, and counteract chemoresistance through different mechanisms, e.g., the regulation of drug transport and metabolism [104]. Several recent reports have shown the use of phytochemicals as efficient chemosensitizers [103].

The role of piperine as a bioavailability enhancer has been clearly depicted. Three main mechanisms have been identified as responsible for piperine’s bioenhancing activity: (i) increment in the blood supply in the enteric vessels; (ii) enhancement of the active transport of drugs across the intestinal barrier; and (iii) inhibition of the enzymes which participate in the inactivation or elimination of drugs [105,106]. Piperine was found to inhibit both drug transporter and drug metabolizing enzymes, thus potentially affecting the plasma concentration of drugs that are substrates of these enzymes [107]. For instance, Li and colleagues [108] demonstrated for the first time that piperine resensitized multidrug-resistant breast and lung tumor cells (MCF-7 and A549, respectively) to doxorubicin through ABC transporter inhibition. At a concentration of 50 µM, piperine lowered the IC_50_ value of doxorubicin by 32- and 14-fold, respectively. Of note, the effect of piperine in reversing P-gp-mediated resistance to doxorubicin was higher in MCF-7 cells than that observed for 50 µM verapamil [108]. Moreover, piperine resensitized MCF-7-resistant cells to mitoxantrone, showing an IC_50_ reduction of mitoxantrone of about seven-fold. Similar results were recorded for P-gp-overexpressing colon and leukemia doxorubicin-resistant cells (CaCO_2_ and CEM/ADR5000), where piperine reverted multidrug resistance [109]. Long-term exposure (48 h stimulation and 24 h recovery) to piperine inhibited the transcription of ABC transporter genes (ABCB1, ABCG2, and ABCC1) [108], suggesting that the downregulation of these efflux pumps may contribute to reverting MDR.

Together with the inhibition of P-gp, piperine contributed to increasing anticancer drugs’ bioavailability, curbing the activity of metabolizing enzymes, such as cytochrome P450 (CYP450) [107]. For instance, piperine inhibited the isoform CYP3A4, which plays a pivotal role in the metabolism of many anticancer drugs, such as docetaxel [110]. The inhibition of CYP3A4 activity by piperine was shown in a xenograft model of human castration-resistant prostate cancer cells, which continue to express androgen-responsive genes, resulting in a more aggressive tumor [111]. In particular, C.B17/Icr-scid PC-3 xenografted mice were orally administered with piperine (100 mg/kg), followed by an intravenous administration of docetaxel (12.5 mg/kg). The group exposed to piperine followed by a docetaxel injection showed the highest increase in the mean plasma concentration of docetaxel, resulting in the most significant inhibition of tumor growth. The rise in the docetaxel plasma concentration did not result in an increase in docetaxel-mediated toxicity [111]. Later studies confirmed the enhancement of docetaxel’s antitumor efficacy in in vitro and in vivo taxane-resistant prostate cancer models [112]. In particular, ICR-NOD/SCID mice implanted with taxane-resistant prostate cancer cells were orally administered 50 mg/kg piperine in association with docetaxel (20 mg/kg). The association of piperine with docetaxel significantly reduced the tumor growth if compared with docetaxel alone and allowed higher intratumor concentrations of docetaxel to be obtained [112]. Of note, the tested concentration of piperine was lower than that in the aforementioned study by Makhov and colleagues [111], due to the severe excitation response that the dose of 100 mg/kg provoked in ICR-NOD/SCID mice [112]. A synergistic interaction between piperine and taxanes was also demonstrated for paclitaxel in breast cancer cells (MCF-7), ovarian cancer cells (SKOV3), and paclitaxel-resistant cervical adenocarcinoma cells (HeLa) [113,114,115].

Since one of the major drawbacks of the usage of piperine is its poor bioavailability, intense research is being carried out to enhance it through pharmaceutical formulation, such as nanoparticles or nanotubes, for piperine alone and in association with anticancer drugs, further boosting the bioavailability of the combination and conceivably its efficacy. In particular, the effects of the association of piperine and rapamycin were assessed using a nanoparticle formulation in breast cancer cells [116]. Pharmacokinetic studies showed a better absorption of the nanoparticles formulated with poly(d,l-lactide-co-glycolide) (PLGA) compared to a rapamycin suspension and a 4.8-fold increase in its bioavailability [116]. Additionally, docetaxel-PLGA micelles tagged with piperine confirmed the 6.5-fold increment in bioavailability compared to the plain drug in neuroblastoma cells [117]. Moreover, the micelles enhanced docetaxel’s cytotoxicity, with a 2.65-fold decrease in the IC_50_ value. The combination of docetaxel plus piperine was also formulated as multiwalled carbon nanotubes. According to previously presented evidence, the pharmacokinetic profile of docetaxel was improved not only by coadministration with piperine, but also by the nanotubes’ formulation [118]. Wistar rats were injected into the tail vein with a 5 mg/kg dose equivalent of docetaxel in nanotubule formulation. The group receiving the nanotubules conjugated along with piperine showed the slowest decline in the docetaxel plasma concentration [118]. Docetaxel nanotubules offered a 2.6-fold enhancement in docetaxel’s bioavailability quantified using AUC. Moreover, the formulation added with piperine produced a 6.4-fold increase compared to the pure drug. The increase in bioavailability was also observed using micelle formulation in BALB/c nude mice xenografted with liver cancer cells (HepG2) [119]. In particular, mixed micelles were delivered through the tail vein in xenografted mice at a dose of 10 mg/kg of docetaxel and 20 mg/kg of piperine. Mixed micelles achieved an increased cytotoxicity and enhanced accumulation in the tumor [119].

Since dysregulation of the apoptotic machinery is involved in the onset of chemoresistance, the ability of piperine to favor apoptosis is an additional mechanism that characterizes its chemosensitizing activity. For instance, piperine enhanced the anticancer effects of tamoxifen, boosting its pro-apoptotic and cytostatic effects in in vitro breast cancer cell models (MCF-7 and T-47D) [30]. Indeed, all the tested IC_50_ fraction combinations resulted in a combination index of about 0.3, which is a value indicating synergistic activity. Furthermore, piperine restored the drug sensitivity to mitomycin-C in resistant cervical cancer cells, inhibiting STAT-3 phosphorylation, nuclear factor kappa-light-chain-enhancer of activated B cells (NF-κB), and Bcl-2 expression and upregulating the activity of the pro-apoptotic proteins Bax, Bid, caspases, and PARP [120]. The anticancer effects of piperine plus mitomycin therapy were demonstrated by the same authors in athymic nude mice xenografted with mitomycin-resistant HeLa cells intraperitoneally treated with piperine (5 mg/kg) and mitomycin (2 mg/mL). A significant suppression of tumor growth was recorded in xenografted mice co-treated with piperine and mitomycin compared to the control or treatment with piperine or mitomycin alone. Immunohistochemical and Western blot analyses demonstrated a decrease in phosphorylated STAT-3-, phosphorylated NF-κB-, and Bcl-2-positive cells, and an increase in caspase-3 and PARP levels, leading to apoptosis in tumor tissues. These results highlighted the synergy of this combination treatment that suppressed tumor growth according to the same pro-apoptotic molecular mechanisms observed in vitro [120].

The apoptotic pathway involving TNF-related apoptosis-inducing ligand (TRAIL) is an attractive target for cancer therapy, due to its high selectivity for cancer cells [121]. To avoid resistance to TRAIL-based therapy, research on compounds enhancing its efficacy represents a promising strategy. Piperine improved the efficacy of TRAIL-based therapy in in vitro and in vivo TRAIL-sensitive breast cancer models [27]. In particular, treatment with 50 mg/kg/day of piperine and an agonistic monoclonal antibody specific for the TRAIL receptor significantly reduced tumor growth in BALC/c female mice orthotopically-inoculated with 4T1 breast cancer cells and to a better extent compared to piperine or a TRAIL monoclonal antibody alone [27].

Despite the promising results reported above, clinical trials are needed to verify the impact of piperine in co-administration with anticancer drugs.

## 4. Other Compounds from *Piper nigrum* with Anticancer Potential

*Piper nigrum* is a source of bioactive molecules with anticancer potential aside from piperine. After piperine, the most investigated pepper alkaloid is piperlongumine, also named piplartine. Piperlongumine represents the main bioactive constituent of long pepper (*Piper longum* L.) and for this reason, it is not extensively covered in the present review. Although piperlongumine was known over 50 years ago, its anticancer activity was only uncovered in the past decade [122]. Piperlongumine shares various anticancer mechanisms with piperine, including the induction of apoptosis, cell-cycle arrest in G1 or G2/M phases, pro-oxidant activity, and anti-metastatic and anti-angiogenic effects. Moreover, piperlongumine synergizes with traditional anticancer drugs [122,123] and exerts selective cytotoxicity towards cancer cells compared to normal ones [123]. Interestingly, a recent paper showed the synergistic effect of the association of piperine plus piperlongumine in triple-negative breast cancer cell lines (MDA-MB-231 and MCF-7) [124]. The synergistic effects of the combination were recorded at the lower doses of the combination (50 or 100 µM piperine with 5 µM piperlongumine), with a selective anticancer effect towards cancer cells compared to normal cells (MCF-10). The pro-apoptotic effect of the combination was independent of the hormone and p53 status, also showing good cytotoxic activity versus MDA-MB-231, which was poorly affected by the cytotoxic effects of piperine alone [124]. This latter result may be due to the fact that the piperine-induced upregulation of Bcl-2 is counteracted by the piperlongumine-induced reduction of this anti-apoptotic gene in this cell line [124].

Pellitorine represents another bioactive compound isolated from *Piper nigrum* showing anticancer activity. Pellitorine from piper roots exerted cytotoxic effects in breast (MCF-7) and human promyelocytic leukemia (HL60) cells, with IC_50_ values of 1.8 and 13 µg/mL, respectively [125].

Although alkaloids are the main component responsible for *Piper nigrum* anticancer effects, the lignan (−)-kusunokinin, isolated from a piperine-free *Piper nigrum* extract, induced anticancer activity in breast (MCF-7, MDA-MB-468, and MDA-MB-231) and colorectal (SW-620) cancer cells [126]. In those cell lines, kusunokinin induced cell-cycle block in the G2/M phase and apoptosis via i) the activation of both the intrinsic and extrinsic pathway; ii) the upregulation of p53, p21, Bax, cytochrome c, caspase-8, caspase-7, and caspase-3; and iii) the downregulation of Bcl-2 [126]. (−)-Kusunokinin showed partial selectivity towards cancer cells compared to normal mammalian cells [126].

Recently, Rattanaburee and colleagues [127] investigated the potential target responsible for the antiproliferative activity of synthetic (±)-kusunokinin. They concluded that the cytostatic effects of this molecule in breast cancer cells relied on its ability to suppress the colony stimulating factor-1 receptor (CSF1R), whose downregulation then affected Akt and its downstream molecules cyclin D1 and CDK1 [127]. Of note, the affinity of the synthetic (±)-kusunokinin for CSF1R is higher than that of natural (−)-kusunokinin, underlying the importance to use these molecules to improve their affinity for the target. A very recent study investigated, for the first time, the anticancer effects of (−)-kusunokinin in vivo [128]. In female Sprague-Dawly rats, mammary tumors were induced through an intraperitoneal injection of 50 mg/kg NMU. (−)-Kusunokinin (7 or 14 mg/kg injected subcutaneously) significantly suppressed tumor growth and no toxic effects were recorded in any of the analyzed organs (heart, liver, lung, spleen, and kidney) or in hematologic and clinical chemistry parameters [128], suggesting a safe profile of this lignan. Furthermore, the study analyzed the anticancer mechanisms of the molecule in breast tumor tissue of treated rats [126]. (−)-Kusunokinin (14 mg/kg) reduced the levels of signaling proteins, i.e., the proto-oncogene tyrosine-protein kinase Src (c-Src), phosphatidylinositol 3-kinases (PI3K), Akt, and p-ERK1/2 and their downstream targets, such as proteins involved in cell-cycle regulation (c-myc, E2F1, CDK1, and cyclin B1) and cell migration (E-cadherin, MMP-2, and MMP-9) [128].

Taken together, these results suggest that piperine is not the only component responsible for the anticancer activity of *Piper nigrum* and that other alkaloids from this *Piper* species, such as pellitorine, piperlongumine, piperlonguminine, and the lignan (−)-kusunokinin, may represent valuable anticancer strategies.

## 5. Toxicological Studies

The acute toxicity of piperine was investigated in mice, rats, and hamsters [129]. The lethal dose causing death in 50% of the dosed animals (LD_50_) values after single intravenous, intraperitoneal, subcutaneous, intragastric, or intramuscular administration to adult male mice were 15.1, 43, 200, 330, and 400 mg/kg b.w., respectively [129]. Lethal dose administration induced animals’ death via respiratory paralysis within 3–17 min [129]. With regards to *Piper nigrum* extract’s acute toxicity, 5000 mg/kg b.w. os of aqueous extract orally administered to male and female Sprague-Dawley rats did not produce any signs of toxicity [130]. Moreover, acute oral toxicity studies of the piperine-free extract PFPE showed no morbidity or mortality up to 14 days in ICR female mice (5000 mg/kg b.w. per os once) and no tissue damage was recorded [19].

Based on the overall weight of evidence, the non-genotoxic nature of piperine was established. The majority of studies exploring the genotoxic potential of piperine have claimed that it has a non-genotoxic nature, when assessed via tests analyzing different genotoxic endpoints [131,132]. In particular, a recent study investigated the in vitro and in vivo genotoxic potential of piperine using the micronucleus test, which allows both aneugenic and clastogenic effects to be detected. No increase in micronuclei was recorded in vitro or in NMRI BR mice exposed up to the maximum tolerated dose of piperine for 2 days (143.5, 287.0, or 574.0 mg/kg b.w. per day; *n* = 10 animals/sex/group) [133]. Furthermore, not only this alkaloid has no genotoxic activity, but it was also able to protect from the genotoxicity of other compounds. As an example, piperine inhibited micronuclei formation, chromosomal aberration, or sister chromatid exchanges induced by different agents, such as aflatoxin B1, cyclophosphamide, mitomycin C, or B(a)P [44,46,134,135,136,137,138,139,140]. The antigenotoxic activity of piperine mainly relies on its ability to (i) inhibit phase I enzymes involved in genotoxicants’ activation and (ii) induce detoxifying enzymes that contrast carcinogens’ activity.

Reproductive toxicity studies are available for piperine [141,142], showing interference with crucial reproductive events. The lowest dose studied (1 mg/kg b.w./day) did not induce any adverse effects on sexual organs and the sperm quality. Doses of 5 mg/kg b.w./day or higher decreased the sexual organs’ weight in male animals and reduced the sperm quality [142,143]. In female animals, oral treatment with 10 or 20 mg/kg b.w. piperine per day up to 14 days decreased the mating performance and fertility index and showed anti-implantation activity 5 days post-mating piperine treatment [141].

A further study investigated the toxic effects of piperine on the liver [144]. After the administration of 1.12 mg/kg b.w./day for 23 days, no histopathological lesions were observed.

Conflicting results are available for the immunomodulative potential of piperine. In early studies performed on Swiss male mice gavaged at 1.12–4.5 mg/kg b.w. per days for 5 days, piperine exhibited immunotoxicity [145]. Treatment at the highest tested dose resulted in a significant decrease in the weight of the spleen, thymus, and mesenteric lymph nodes and caused a significant reduction in total leucocytes [145]. At 2.25 and 4.5 mg/kg, piperine inhibited the response of B lymphocytes to the mitogenic stimulus. The lowest dose (1.12 mg/kg) was devoid of immunotoxic effects and was identified as the no observed adverse effect level (NOAEL) for this effect [144]. However, piperine exhibited a protective effect against cadmium-induced immunotoxicity [146].

Recently, the European Food Safety Authority (EFSA) identified the NOAEL of piperine, which is 5 mg/kg b.w. per day based on the most comprehensive study available (90-day dietary toxicity study in rats) [147].

No experimental carcinogenicity studies are available for piperine. However, in silico models predicted a non-carcinogenic effect for piperine [148].

A NOAEL value was established for piperine, as reported above. However, there are conflicting results and missing information, in particular for its reprotoxic effects, that make the NOAEL value uncertain. For this reason and considering that piperine is not genotoxic, an approach based on the Threshold of Toxicological Concern (TTC) has been used. According to its structure, piperine is a Cramer Class III compound [149]. The Cramer Class III TTC threshold was found to be 1.5 μg/kg b.w./day [150].

Although the dietary consumption of black pepper varies considerably within the population, EFSA calculated that the estimated exposure to piperine from natural sources when consuming black pepper as a flavoring ingredient is 6.2 µg/day in Europe and 0.07 µg/day in the USA, based on the maximized survey-derived daily intake [147], which are below the TTC threshold level of 1.5 μg/kg b.w./day (90 μg/day) for Cramer Class III compounds.

In 2016, the Norwegian Scientific Committee for Food Safety (VKM) was required to assess the risk derived from piperine daily intake through food supplements, which was estimated to be 1.5 mg/day by the Norwegian Food Safety Authority. VKM concluded that the daily dose of 1.5 mg piperine in food supplements is unlikely to cause adverse health effects in children, adolescents, or adults, based on the margin of exposure approach (ratio of the NOAEL to the exposure) [150].

Of note, the doses of piperine used in the in vivo anticancer studies are higher than the calculated NOAEL and TTC values. Taken together, data on the putative toxicities of piperine at doses eligible for anticancer activity and after long periods of administration are not exhaustive. A risk/benefit evaluation is still required to figure out its potential use as an anticancer strategy.

## 6. Conclusions

**Figure 1:**
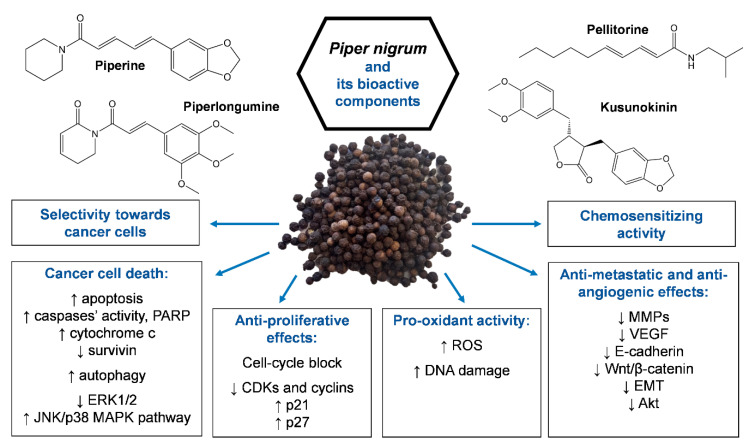
Anticancer activity of *Piper nigrum* and its bioactive components. ↑: increase; ↓: decrease; PARP: poly(ADP-ribose) polymerase; ERK1/2: extracellular signal-regulated kinase 1/2; MAPK: mitogen-activated protein kinase; CDKs: cyclin-dependent kinases; MMPs: matrix metalloproteinases; VEGF: vascular endothelial growth factor; and EMT: epithelial mesenchymal transition.

*Piper nigrum* is one of the most popular spices in the world, with a day-to-day use and growing fame as a source of bioactive molecules with pharmacological properties. *Piper nigrum* has been reported to possess undeniable anticancer potential in different cancer cell lines and animal models (Figure 1). Although piperine is the major active constituent of black pepper and the most characterized in its multiple mechanisms of action counteracting cancer development, other constituents, such as piperlongumine, pellitorine, and kusunokinin, have been demonstrated to have remarkable anticancer properties. Interestingly, among *Piper nigrum* extracts, a piperine-free preparation exhibited higher anticancer activity in vitro and in vivo compared to piperine [18,19,20], highlighting the synergistic anticancer activity of the different *Piper nigrum* components aside from piperine.

The ability of piperine to modulate certain metabolizing enzymes, together with its capacity to inhibit efflux transporters, i.e., Pgp, and its pro-apoptotic activity are at the basis of piperine’s bioenhancing properties. Indeed, the combination of piperine with traditional anticancer drugs results in a promising enhancement of their bioavailability and efficacy, together with the restoration of chemosensitivity in several in vitro and in vivo models. However, caution is required for the translation of these interesting properties in medical practice, mainly due to potential drug interactions [111,151] and the low bioavailability of piperine [98,99]. The optimization of a proper dose-regimen and of pharmaceutical formulations is a necessary step for exploring the therapeutic potential of piperine for cancer patients.

Despite the outstanding potential of piperine reported in pre-clinical studies [152], no clinical trials are ongoing in cancer patients. The bioenhancing properties of piperine are being explored in a clinical trial in association with curcumin to assess whether the combination may reduce inflammation and discomfort from a ureteric stent in cancer patients (ClinicalTrials.gov Identifier: NCT02598726).

The synergistic or additional effects arising from the combination of chemotherapeutic agents and phytochemicals, of which *Piper nigrum* is rich, represent a cutting-edge topic for anticancer research. However, human studies and clinical trials deepening the bioenhancing properties and anticancer effects of *Piper nigrum* components alone and in association with anticancer drugs are missing, although crucial for supporting the efficacy and safety in cancer patients.

## Figures and Tables

**Table 1:** In vitro and in vivo anticancer effects of *Piper nigrum* extracts.

*Piper nigrum* Extracts	Experimental Model	IC_50_^a^ or EC_50_^b^ (Time of Treatment)	Anticancer Effects and Molecular Targets	Reference
Seeds’ ethanolic extract (50% ethanol)	Colorectal cancer cells (HCT-116, HCT-15, HT-29)	IC_50_: HCT-116: 4.0 (24 h) 3.1 (48 h) 3.4 (72 h) μg/mL HCT-15: 3.2 (24 h) 2.9 (48 h) 1.9 (72 h) μg/mL HT-29: 7.9 (24 h) 6.1 (48 h) 7.4 (72 h) μg/mL	↑ tumor cell death	[13]
Seeds’ extract, SnO_2_ nanoparticles	Colorectal cancer cells (HCT-116) and lung cancer cells (A549)	IC_50_: HCT-116: 165 µM A549: 135 µM	↑ ROS ^c^	[14]
Fruits’ ethanolic extract	Vitro: Breast cancer cells (MCF-7) and colon cancer cells (HT-29) (1–1000 µg/mL) Vivo: Ehrlich ascites carcinoma-bearing male Balb/c mice (intraperitoneal injection (i.p.), 100 mg/kg/day in saline containing 1% Tween 80, for 9 days)	EC_50_: MCF-7: 27.1 µg/mL (24 h) HT-29: 80.5 µg/mL (24 h)	Vitro: ↑ tumor cell death ↓ tumor cell proliferation ↑ ROS, ↑ DNA damage Vivo: ↓ tumor growth, ↑ mice survival ↑ apoptosis cell-cycle arrest at G1/S (↑ Bax, p53; ↓Bcl-xL, cyclin A) ↑ oxidative stress (↑ lipid peroxidation, protein carbonylation, GR ^d^, SOD ^e^, CAT ^f^)	[15]
Supercritical fluid extract (SFE) of fruits’ ethanolic extract	Vitro: Breast cancer cells (MCF-7) (1–1000 µg/mL) Vivo: Ehrlich ascites carcinoma-bearing male Balb/c mice (i.p., 10 or 100 mg/kg/day in saline containing 1% Tween 80, for 9 days)	EC_50_: 14.40 μg/mL (72 h) IC_50_: 27.8 μg/mL (24 h)	Vitro: ↑ apoptosis Silico (docking study): Piperine interaction with CDK2 ^g^, ATP binding site; cyclin A binding site and Bcl-xL binding site. Vivo: ↓ tumor growth, ↑ mice survival ↑ apoptosis cell-cycle arrest at G2/M (↑ Bax, p53; ↓Bcl-xL, ↓cyclin A, ↓CDK2)	[16,17]
Fruits’ (i) methanol crude extract or (ii) dichloromethane crude extract	Breast cancer cells (MCF-7, MDA-MB-231, MDA-MB-468)	IC_50_ (72 h) methanol crude extract: MCF-7: 20.25 µg/mL MDA-MB-231: 22.37 µg/mL MDA-MB-468: 9.04 µg/mL IC_50_ (72 h) dichloromethane crude extract: MCF-7: 23.46 µg/mL MDA-MB-231: 38.82 µg/mL MDA-MB-468: 7.94 µg/mL	↑ tumor cell death	[18]
Piperine-free *Piper nigrum* fruits’ extract (PFPE)	Vitro: Breast cancer cells (MCF-7, MDA-MB-231, MDA-MB-468, ZR-75-1), colorectal cancer cells (HT-29, SW-620), lung cancer cells (H358, A549), neuroblastoma cells (LA-N-5, SK-N-SH). Vivo: Female ICR mice (oral administration (os) 5000 mg/kg b.w. in mixture of distilled water and Tween-80 (4:1 *v*/*v*) for acute oral toxicity studies) or NMU-treated female Sprague-Dawley treated orally with (i) 100 or 200 mg/kg b.w. in mixture of distilled water and Tween-80 (4:1 *v*/*v*) at 14 days after NMU application three times per week up to 76 days, or (ii) 100, 200, or 400 mg/kg b.w. PFPE after the first NMU-induced tumor every two days up to 30 days	IC_50_ (72 h): MCF-7: 7.45 µg/mL MDA-MB-231: 22.67 µg/mL MDA-MB-468: 18.19 µg/mL ZR-75-1: 13.85 µg/mLHT-29: 27.74 µg/mL SW-620: 29.56 µg/mL H358: 34.69 µg/mLA549: 30.77 µg/mL LA-N-5: 111.28 µg/mL SK-N-SH: 21.51 µg/mL	Vitro: ↓ cell proliferation ↑ apoptosis (↑ p53 and cytochrome c; ↓ topoisomerase II) Vivo: ↓ tumor bearing rats ↓ tumor size, ↑ cytochrome c in tumor tissues	[19]
Piperine-free *Piper nigrum* fruits’ extract	Vitro: Breast cancer cells (MCF-7) Vivo: NMU-treated female Sprague-Dawley rats. PFPE treatment regimen as previously described above		Vitro: ↓ E-cadherin, c-myc, VEGF ^h^ Vivo: ↑ p53 ↓ E-cadherin, MMP ^i^-9, MMP-2, c-myc, and VEGF	[20]
Root dried power crude (i) petroleum ether extract, (ii) chloroform extract, (iii) ethylacetate extract	Promyeolocytic leukemia cells (HL60)	IC_50_: petroleum ether extract (72 h): 11.2 µg/mL chloroform extract (72 h): 9.8 µg/mL ethylacetate extract (72 h): /	↑ tumor cell death	[21]

↑: increase; ↓: decrease; ^a^ IC_50_: half maximal inhibitory concentration; ^b^ EC_50_: half maximal effective concentration; ^c^ ROS: reactive oxygen species; ^d^ GR: glutathione reductase; ^e^ SOD: superoxide dismutase; ^f^ CAT: catalase; ^g^ CDK2: cyclin-dependent kinase 2; ^h^ VEGF: vascular endothelial growth factor; and ^i^ MMP: matrix metalloproteinase.

**Table 2:** In vitro and in vivo anticancer activity of piperine.

Cancer Type	Experimental Models	Piperine	IC_50_^a^	Anticancer Effects and Molecular Targets	Reference
Breast cancer	Vitro: 4T1 mouse mammary carcinoma cells Vivo: Female BALB/c mice syngeneic to 4T1 cells (4T1 cells transplanted subcutaneously)	Vitro: 35–280 µM Vivo: Intratumoral injection of 2.5 or 5 mg/kg every 3 days 3 times	105 ± 1.08 µM (48 h) 78.52 ± 1.06 µM (72 h)	Vitro: ↑ apoptosis (↑ caspase-3 activity) ↓proliferation (↓ cyclin B1, cell-cycle block in G2/M phase) ↓ migration; ↓ MMP ^b^-9 and MMP-13 Vivo: ↓ tumor growth ↓ lung metastasis	[25]
	HER-overexpressing cells: SKBR3 and BT-474 Basal HER-expressing cells: MCF-7 and MDA-MB-231	10–200 µM	SKBR3 50 µM (48 h) MCF-7 > 200 µM (48 h)	↑ apoptosis (↑ caspase-3 activity, cleaved-PARP ^c^, DNA damage) ↓ HER2 ^d^ expression ↓ SREBP-1 ^e^ and fatty acid synthase via ERK1/2 ^f^ inhibition ↓ MMP-9 via inhibition of Akt and MAPK ^g^ signaling	[26]
	Vitro: MDA-MB-231, MDA-MB-468, murine 4T1 Vivo: BALB/c female mice orthotopically-inoculated 4T1	Vitro: 25–200 µM Vivo: Oral administration (os) 50 mg/kg/day from day 7 to 21		Vitro: ↓ proliferation (cell-cycle block in G2/M phase) ↓ survivin and p65 phosphorylation Vivo: ↓ tumor growth	[27]
	MDA-MB-231, MDA-MB-468, T-47D, and MCF-7	50–150 µM		↑ apoptosis (↑ Smac/DIABLO ^h^, cytochrome c; ↓ IAPs ^i^) ↓ cell-cycle progression (↑ p21; ↓ CDK ^j^4, CDK1, cyclin D3, cyclin B, E2F1 ^k^, CDC25 C ^l^) ↓ mammospheres’ growth ↓ MMP-2, MMP-9	[28]
	Vitro: Mouse mammary EMT6/P cancer cells Vivo: Balb/C female mice with EMT6/P cells injected subcutaneously in the abdominal area	Vitro: 50–1200 µM Vivo: Intraperitoneal injection (i.p.) 25 mg/kg/day in PBS for 14 days	870 µM (48h)	Vitro: ↑ apoptosis (↑ caspase-3 activity) ↓ VEGF ^m^ Vivo: ↓ tumor size ↑ apoptosis in tumor tissue ↓ ALT ^n^, AST ^o^, creatinine	[29]
	MCF-7, T-47D	3–100 µM	MCF-7 37.34 µM (24 h) T-47D 61.05 µM (24 h)	↑ apoptosis (↑ Bax, ↓ Bcl-2) ↓ proliferation (cell-cycle block in G2/M phase)	[30]
	MDA-MB-231	20–320 µM	238 µM (72 h)	↓ proliferation	[31]
Prostate cancer	DU145, LNCaP, and PC3	20–320 µM	LNCaP 74.4 µM (24 h) DU145 226.6 µM (24 h) PC3 111.0 µM (24 h)	↓ proliferation (cell-cycle block in G0/G1 phase, ↓cyclin D1 and cyclin A; ↑ p21 and p27) ↑ autophagy (↑ LC3B ^p^-II and LC3B puncta formation)	[32]
	Vitro: DU145, LNCaP, 22RV1, and PC3 Vivo: Nude mice (LNCaP or DU145 transplanted subcutaneously)	Vitro: 50–200 µM Vivo: I.p., 100 mg/kg/day in vegetable oil for 1 month os 10 mg/kg body weight (b.w.) for 1 month	LCNaP 60 µM (24 h) PC3 75 µM (24 h) 22Rv1 110 µM (24 h) DU145 160 µM (24 h)	Vitro: ↑ apoptosis (↑ caspase-3 activity and cleaved-PARP) ↓ migration (↓ STAT-3 ^q^ and NF-kB ^r^) Vivo: ↓ tumor growth	[33]
	LNCaP and PC3	5–150 µM	LNCaP 39.91 µM (24 h) PC3 49.45 µM (24 h)	↑ apoptosis ↓ proliferation (cell-cycle block in G0/G1) via voltage-gated K^+^ current blockade	[34]
	LNCaP ad PC3	0.1–100 µM	LNCaP 39.91 µM (24 h) PC3 49.45 µM (24 h)	↑ apoptosis ↓ proliferation (cell-cycle block in G1 phase) via voltage-gated K^+^ current inhibition	[35]
	DU145	80–320 µM		↑ apoptosis (↑ Bax, ↓ Bcl-2) ↓ proliferation ↓ migration (↓ MMP-9 via inhibition of Akt/mTOR signaling)	[36]
Colon cancer	DLD1	1–200 µM		↓ proliferation	[37]
	HT-29, Caco-2, SW480, HCT-116 (p53+/+), and HCT-116 (p53−/−)	10–150 µM	HT-29 53 ± 1 µM (72 h) Caco-2 54 ± 5 µM (72 h) SW480 126 ± 3 µM (72 h) HCT-116 (p53+/+) 109 ± 9 µM (72 h) HCT-116 (p53−/−) 118 ± 7 µM (72 h)	↑ apoptosis (↑ loss of mitochondrial membrane potential, caspase activity, cleaved-PARP) ↑ ROS ^s^ ↑ endoplasmic reticulum stress (↑ IRE1α ^t^, CHOP ^u^, BiP ^v^) ↓ survivin ↓ proliferation (cell-cycle block in G1 phase; ↓ cyclin D1 and cyclin D3, CDK4 and CDK6; ↑ p21 and p27) ↓ colony formation and spheroids’ growth	[38]
	HCT6, SW480, and DLD1	20–200 µM		↓ proliferation ↓ migration ↓ Wnt/β-catenin and GSK3β ^w^	[39]
	SW480 and HCT-116	25–800 µM		↓ migration and EMT ^x^ (↓ STAT-3/Snail, ↓ vimentin, ↑ E-cadherin)	[40]
Rectal cancer	HRT-18	10–150 µM		↑ apoptosis ↓ proliferation (block cell-cycle progression) ↑ ROS	[41]
Lung cancer	Vivo: C57BL/6 Mice lung metastasis from melanoma cells (B16F-10 lateral tail vein injection)	I.p., 200 μmol/kg b.w. in 0.1% gum acacia for 10 days		↑ animal survival ↓ metastatic lung fibrosis, ↓ uronic acid and hexosamine in lung tissue ↓ serum level of sialic acid and GGT ^y^	[42]
	Vivo: Swiss Albino mice benzo(a)pyrene induced lung cancer (os in corn oil 50 mg/kg b.w.)	Os 50 mg/kg b.w. in corn oil: (i) On alternate days for 16 weeks immediate after the first dose of carcinogen; (ii) piperine as (i), but starting from the sixth week of B(a)P till the end of the experiment		↓ lipid peroxidation, protein carbonyls, nucleic acid content, and polyamine synthesis in lung	[43]
	Vivo: Swiss Albino mice benzo(a)pyrene induced lung cancer (os in corn oil 50 mg/kg b.w.)	Os 50 mg/kg b.w. in corn oil for 16 weeks. Treatment: (i) Immediately after the first dose of benzo(a)pyrene; (ii) after the last dose of benzo(a)pyrene		↓ hexose, hexosamine and sialic acid in serum, liver, and lung tissues	[44]
	A549	25–400 µM	122 µM (48 h)	↑ apoptosis (↑ caspase3 and -9 activity, Bax/Bcl-2 ratio, p53 expression) ↓ Proliferation (cell-cycle block in G2/M phase)	[45]
	A549	100–500 µM		↑ apoptosis (↓ c-myc)	[46]
	A549	20–320 µM	198 µM (72 h)	↓ EMT (↓ fibronectin and N-caderin, ↑ E-cadherin) ↓ ERK 1/2 and SMAD ^z^ 2 ↓ migration (↓ MMP-2)	[31]
Melanoma	SK MEL 28, A375 (human cells), and B16 F0 (murine cells)	75–300 µM	SK MEL 28 221 µM (24 h) 172 µM (48 h) 136 µM (72 h) B16 F0 200 µM (24 h) 155 µM (48 h) 137 µM (72 h) A375 225 µM (24 h) 160 µM (48 h) 100 µM (72 h)	↑ apoptosis (↑ p53; ↓ XIAP ^aa^, Bid ^ab^; ↑ Caspase-3 and cleaved-PARP) ↓ proliferation (cell-cycle block in G1 phase; ↓ cyclin D, E2F1, and Rb ^ac^ phosphorylation; ↑ p21, ATR ^ad^, Chk ^ae^ 1) ↑ ROS ↑ DNA damage (↑ H2AX ^af^ phosphorylation)	[47]
	Vitro: A375SM (highly metastatic), A375P (moderately metastatic) Vivo: BALB/c nude mice (nu/nu) (A375SM or A375P transplanted subcutaneously)	Vitro: 50–200 µM Vivo: Os 50 or 100 mg/kg b.w. in water 5 times per week for 4 weeks		Vitro: ↑ apoptosis (↑ Bax, cleaved-PARP, caspase-9, ↓ Bcl2) ↑ JNK/p38 MAPK phosphorylation, ↓ ERK1/2 Vivo: ↓ tumor growth ↑ apoptosis (↑ caspase-3) ↓ ERK1/2	[48]
Hepatocellular cancer	Vitro: HepG2 Vivo: Male Wistar rats tumor induced using diethylnitrosamine (DEN, 0.01% of DEN in drinking water for 16 weeks)	Vitro: 5–100 µM Vivo: Os 5 mg/kg b.w. in corn oil for 6 weeks starting from the 10th week of the experimental period	75 µM (24 h) 30 µM (48 h)	↑ apoptosis (↑ cleaved caspase-3 and caspase-9, mitochondrial permeabilization, Bax, cytochrome c release, ↓ Bcl-2) ↓ proliferation ↑ ROS (↓ catalase) ↓ ERK1/2 and SMAD Vivo: ↓ AST, ALP ^ag^, and ALT ↑ improvement in liver architecture ↓ Ki67	[49]
	HepG2	20–320 µM	214 µM (72 h)	↓ proliferation	[31]
Ovarian	A2780	4–20 µM		↑ apoptosis (↑ cytochrome c release, caspase-3 and caspase-9 activity, cleaved-PARP) ↑ JNK and p38 MAPK phosphorylation	[50]
	OVACAR-3 (ovarian cisplatin-resistant cells)	3.12–200 µM	28 µM (24 h)	↑ apoptosis (↑ caspase-3, caspase-9, and Bax) Cell-cycle block in G2/M phase ↓ migration ↓ MAPK signaling (PI3 K ^ah^/Akt/GSK3β)	[51]
Osteosarcoma	HOS and U2OS	25–200 µM	HOS 72 µM (72 h) H2OS 126 µM (72 h)	↓ proliferation (cell-cycle block in G2/M phase, ↓ cyclin B1, ↑ CDK1, Chk2) ↓ Akt, ↑ c-JNK/p38 MAPK phosphorylation ↓ migration (↓ MMP-2 and MMP-9; ↑ TIMP1/2 ^ai^)	[52]
	U2OS and 143B	50–150 µM		↓ cell proliferation ↑ apoptosis ↓ invasion and angiogenesis (↓ MMP-2 and VEGF) ↓ Wnt/β-catenin and GSK3β (↓ cyclin D1, c-Myc, and COX-2 ^aj^)	[53]
Fibrosarcoma	HT-1080			↓ MMP-9	[54]
Oral squamous carcinoma	KB	25–300 µM	124 µM (24 h)	↑ apoptosis (↑ caspase-3 activity, loss mitochondrial potential) ↑ ROS ↓ proliferation (cell-cycle arrest in G2/M phase)	[55]
Cervical adenocarcinoma	HeLa	10–200 µM		↑ apoptosis (↑ caspase-3 activity, loss mitochondrial potential) ↑ ROS ↑ DNA damage ↓ proliferation (cell-cycle arrest in G2/M phase)	[56]
Leukemia	HL60	10–200 µM	25 µM (24 h)	↑ apoptosis (↑ Bax, ↓ Bcl-2) ↑ autophagy ↓ cell proliferation (cell-cycle arrest in S phase) ↓ migration	[57]

↑: increase; ↓: decrease; ^a^ IC_50_: half maximal inhibitory concentration; ^b^ MMP: matrix metalloproteinase; ^c^ PARP: poly(ADP-ribose) polymerase; ^d^ HER2: human epidermal growth factor receptor 2; ^e^ SREBP-1: sterol regulatory element-binding protein-1; ^f^ ERK1/2: extracellular signal-regulated kinase 1/2; ^g^ MAPK: mitogen-activated protein kinase; ^h^ Smac/DIABLO: second mitochondria-derived activator of caspase/direct inhibitor of apoptosis protein (IAP)-binding protein with low PI; ^i^ IAPs: inhibitors of apoptosis proteins; ^j^ CDK: cyclin-dependent kinase; ^k^ E2F1: E2F Transcription Factor 1; ^l^ CDC25C: cell division cycle 25C; ^m^ VEGF: vascular endothelial growth factor; ^n^ ALT: alanine transaminase; ^o^ AST: aspartate transaminase; ^p^ LC3B: microtubule-associated protein 1A/1B-light chain 3—phosphatidylethanolamine conjugate; ^q^ STAT-3: signal transducer and activator of transcription 3; ^r^ NF-kB: nuclear factor kappa-light-chain-enhancer of activated B cells; ^s^ ROS: reactive oxygen species; ^t^ IRE1α: inositol-requiring 1α; ^u^ CHOP: C/EBP homologous protein; ^v^ Bip: binding immunoglobulin protein; ^w^ GSK3β: glycogen synthase kinase 3β; ^x^ EMT: epithelial mesenchymal transition; ^y^ GGT: gamma glutamyl traspeptidase; ^z^ SMAD: small mother against decapentaplegic; ^aa^ XIAP: human X-linked IAP; ^ab^ Bid: BH3 interacting domain death agonist; ^ac^ Rb: retinoblastoma protein; ^ad^ ATR: ataxia telengectasia and Ras3-related protein; ^ae^ Chk: checkpoint kinase; ^af^ H2AX: H2A histone family member X; ^ag^ ALP: alkaline phosphatase; ^ah^ PI3K: phosphatidylinositol 3-kinases; ^ai^ TIMP1/2: inhibitors of metalloproteinase 1/2; and ^aj^ COX-2: cyclooxygenase-2.

**Table 3:** Selectivity of piperine towards cancer cells.

Experimental Model	Treatment Conditions	Selectivity (Compared to Cancer Cell Lines)	Reference
Murine fibroblasts (NIH3T3)	IC_50_^a^ 232 ± 1.15 µM (48 h)	+ (4T1)	[25]
Human normal prostate epithelial cells (RWPE-1)	<160 µM (48 h)	+ (LNCaP, DU145, PC3)	[32]
Human lung fibroblasts (WI38)	25–400 µM (48 h)	++ (A549)	[45]
Human osteoblasts (hFOB)	25–200 µM (72 h)	+ (HOS, U2OS)	[52]
Fibroblasts and human mammary epithelial cells	50–150 µM (72 h)	++ [Caco-2, SW480, HCT-116 (p53+/+), HCT-116 (p53−/−)]	[38]
Human mammary epithelial cells	50–150 µM (72 h)	+ (MDA-MB-231, MCF-7, T-47D, MDA-MB-468)	[28]
Primary monolayer cultures of adult rat hepatocytes	Up to 100 µM (48 h)	++ (HepG2)	[49]
Human normal ovarian cells (OSE)	0–20 µM (72 h)	+ (A2780)	[50]
Human intestinal cells (IEC-6)	20–200 µM (24, 48, 72 h)	++ (HCT-116, SW480, DLD1)	[39]

^a^ IC_50_: half maximal inhibitory concentration; +: selectivity; and ++: high selectivity.
